# Developing and comparing a new BMI inclusive energy expenditure algorithm on wrist-worn wearables

**DOI:** 10.1038/s41598-025-99963-0

**Published:** 2025-06-19

**Authors:** Boyang Wei, Christopher Romano, Mahdi Pedram, Bonnie Nolan, Whitney A. Morelli, Nabil Alshurafa

**Affiliations:** 1 Department of Preventive Medicine, Northwestern University, Chicago, 60611 USA; 2 Department of Computer Science, Northwestern University, Evanston, 60208 USA; 3 Department of Computer Science & Engineering, University of North Texas, Denton, 76203 USA; 4 Department of Physical Medicine and Rehabilitation, Medical College of Wisconsin, Milwaukee, 53226 USA

**Keywords:** Actigraphy, Machine learning, Metabolic equivalent of task, Obesity, Translational research, Software

## Abstract

Estimating energy expenditure (EE) in real-world settings is crucial for studying human behavior and energy balance. Despite advances in wrist-worn inertial measurement units (IMU), actigraphy remains the most accepted measure for estimating EE, despite known Errors in accuracy, particularly in people with obesity. We developed an algorithm estimating EE from commercial smartwatch sensor data, and validated it against actigraphy-based energy estimates in people with obesity. In an in-lab study, 27 participants wore a Fossil Sport smartwatch and ActiGraph wGT3X+ while performing activities of varying intensities. Another 25 participants wore the smartwatch for 2 days in a free-living study. We built a machine learning model to estimate metabolic equivalent of task (MET) values/minute using smartwatch accelerometer and gyroscope data. Analysis included 2,189 minutes of in-lab and 14,045 minutes of free-living data. Compared to the metabolic cart, our model achieved lower root mean square error (0.28–0.32) across various sliding windows. In the free-living study, our algorithm’s estimates fell within $\pm 1.96$ SD of the best actigraphy-based estimates for 95.03% of minutes. Our proposed method accurately estimated METs compared to 11 algorithms primarily validated in non-obese populations, suggesting that commercial wrist-worn devices can provide more inclusive and reliable EE measures using our algorithm.

## Introduction

Wearable devices present new opportunities to study physical activity and energy expenditure (EE) by enabling continuous, minimally obtrusive physiologic measurements to be performed in non-laboratory settings. Wearables have become prominent in developing and deploying behavioral interventions and physical activity surveillance systems and are commonly used to estimate EE. The use of body-worn activity monitors to obtain objective measures of free-living EE has shown considerable promise, owing to these devices’ relative ease of use and reasonable accuracy^1,2^. However, established body-mounted sensors currently yield highly variable estimates of EE in specific populations^3^. Specifically, estimates using hip-worn ActiGraph activity monitors (ActiGraph Corp, Pensacola, FL) have been shown to overestimate physical activity (PA) and EE for moderate-intensity activities^4^. However, recent research shows minor differences in accelerometry measurements when sensors are worn on different body locations^2^, such as hip- and wrist-mounted devices^5^. A recent review suggests that, although the hip provides a more accurate estimate of EE, the accuracy gap between wrist- and hip-worn actigraphy is narrowing^6^. Despite this, hip-worn actigraphy remains the most commonly used measure of EE in free-living people^7^.

Among free-living people, people with obesity stand to benefit immensely from physical activity trackers. However, they exhibit known differences in walking gait and postural control^8^, resting EE^9^, preferred walking speed^10^, and physical function compared to people without obesity^11^. Hip-worn devices are prone to decreased accuracy in people with obesity due to biomechanical differences such as altered gait patterns and device tilt angle^12^. These factors can lead to inconsistent and less reliable measurements of physical activity and energy expenditure. In contrast, wrist-worn devices may overcome some of these limitations by improving adherence^13^ and being less impacted by body composition variations^14^. The increased comfort and convenience of wrist-worn devices make them more appealing for continuous wear, which is crucial for capturing a comprehensive picture of daily physical activity. Despite these potential advantages, wrist-worn devices have not been extensively validated within this population^15,16^. Currently, there is no algorithm that researchers can use to estimate EE reliably from commercial wrist-worn devices that has been validated in people with obesity.

Adherence to using wearable sensors is a major barrier to studying free-living human behavior^13^. Given the popularity and widespread availability of commercial wrist-mounted sensors, the use of commercial sensors in lieu of research-grade sensors presents many advantages and opportunities. However, the accuracy and validity of EE estimates from commercial wrist-mounted sensors compared to research-grade sensors^17^ and related algorithms are heavily understudied. Existing commercial wrist-mounted device companies have developed algorithms to determine calorie expenditure; however, these algorithms remain proprietary and lack transparency in their validation. Thus, an open-access algorithm and open-sourced dataset that researchers can use to validate EE from commercial wrist-mounted devices is critical to improve our understanding of EE in free-living people with obesity.

Given the high variability in concordance between gross body movement and EE, especially at the wrist, advanced machine learning algorithms are increasingly being deployed to estimate EE and classify physical activities^18–20^. It is well-known that personalized machine learning algorithms are best suited for use in the population they were trained on^21,22^, achieving higher prediction accuracy by adapting to the characteristics of the individuals. Earlier studies have demonstrated that grouping activities of the same type together, regardless of their intensity level, before creating a regression model can improve EE estimates by reducing variability^23^. In this paper, we present a machine-learning model to estimate minute‑by‑minute metabolic equivalent of task (MET) values from smartwatch accelerometer and gyroscope data, benchmarked against four wrist‑ and seven hip‑based ActiGraph algorithms under laboratory conditions, and evaluated against the top‑performing algorithm in free‑living individuals with obesity.

## Results

### Participants

We enrolled 27 participants (17 female) with obesity for the in-lab study and 25 participants with obesity (16 female) for the free-living study. See Table 1 for descriptive statistics of participant age, height, weight, and BMI for both studies. In total, we analyzed data from 52 participants, including 1,838 minutes of in-lab data and 14,045 minutes of free-living data. Participants were free to remove the camera device from the body at any time or turn it off at their own discretion for privacy concerns in the free-living study. Any data the participants collected that they were no longer comfortable sharing with the research team was deleted. We also removed 87 minutes of free-living data from our analysis because of obstruction of the wearable camera (and therefore loss of behavioral ground truth)^24^.

**Table 1:** Descriptive statistics of all 52 participants across in-lab and free-living studies.

			Mean	Std	Median
		**Age**	44.2	12.1	45.0
**In-lab**	**Female**	**Height (inches)**	64.3	2.27	64.0
	(n=17)	**Weight (lbs)**	221.8	32.4	222.0
		**BMI**	37.6	4.54	38.4
		**Age**	41.3	10.61	42.5
**In-lab**	**Male**	**Height (inches)**	69.9	2.98	69.8
	(n=10)	**Weight (lbs)**	249.1	32.81	245.8
		**BMI**	35.9	4.26	34.2
		**Age**	43	12	41
**Free-living**	**Female**	**Height (inches)**	65	2	65
	(n=16)	**Weight (lbs)**	209	36	201
		**BMI**	34	5	34
		**Age**	51.2	14.7	57
**Free-living**	**Male**	**Height (inches)**	70.3	2.9	70
	(n=9)	**Weight (lbs)**	258.2	23.8	258
		**BMI**	36.3	3.77	37

BMI, body mass index.

**Table 2:** METs estimation root mean square error (RMSE) by different window sizes and activity types.

Window (s)	Sedentary	Light	Moderate/Vigorous	Overall
5	0.321	0.361	0.352	0.336
10	0.287	0.399	0.354	0.316
12.8	0.320	0.440	0.306	0.333
15	0.260	0.589	0.319	0.326
30	0.285	0.445	0.296	0.310
40	0.280	0.421	0.318	0.307
50	0.280	0.312	0.286	0.298
**60**	**0.268**	**0.307**	**0.310**	**0.281**
90	0.290	0.316	0.310	0.295

### In-lab MET evaluation

We enrolled 27 participants in the in-lab study. One participant was excluded due to data collection equipment failure resulting in missing data. In comparing predicted METs to ground truth METs derived from the metabolic cart, we experimented with 9 window sizes ranging from 5 to 90 seconds. Our proposed method achieved best performance at the 60-second window length (Table 2). The estimation of METs from the regression model returned a 0.281 RMSE across sedentary, light, and moderate- to-vigorous activities (Table 2). The XGBoost binary classifier demonstrated strong performance in distinguishing between sedentary and non-sedentary activities, achieving 0.952 precision, 0.628 recall, and 0.758 F1-score (Table 3). We also found that among methods using the 60-second window size^7,17,25^, our proposed method yielded the lowest RMSE of 0.281, and Kerr et al.’s method^7^ yielded the second lowest RMSE of 0.317 (Table 4). Our recall is lower than our precision because certain sedentary activities involve wrist movement (e.g., phone use or gesturing), which may lead the classifier to incorrectly label them as non-sedentary. However, our algorithm mitigates this limitation by applying a regression model in Stage 2 to all windows classified as non-sedentary, which allows the MET estimate to reflect the true intensity based on extracted features. Therefore, even if the classifier incorrectly classifies a sedentary activity, the resulting MET value remains close to ground truth, minimizing errors on overall energy expenditure estimation.

**Table 3:** Classification performance metrics of sedentary activity by different window sizes corresponding to related works.

Window (s)	Precision	Recall	F1-score
10	0.957	0.682	0.797
12.8	0.955	0.698	0.807
15	0.951	0.624	0.754
30	0.961	0.692	0.805
60	0.952	0.628	0.758

**Table 4:** METs estimation root mean square error (RMSE) by proposed and related works.

Method	Location	Window	Sedentary	Light	Moderate/Vigorous	Overall
Crouter^32^	Hip	**10**	0.446	0.842	0.460	0.515
**Proposed**	**Wrist**		**0.287**	**0.399**	**0.354**	**0.316**
Ray^26^	Hip	**12.8**	0.277	0.412	0.302	0.301
**Proposed**	**Wrist**		**0.320**	**0.440**	**0.306**	**0.333**
Staudenmayer^31^	Wrist	**15**	0.366	0.573	0.316	0.386
**Proposed**	**Wrist**		**0.260**	**0.589**	**0.319**	**0.326**
Montoye^30^	Wrist	**30**	0.264	0.734	0.633	0.436
Montoye^34^	Hip		0.262	0.576	0.415	0.345
Lyden^33^	Hip		0.260	0.701	0.224	0.335
**Proposed**	**Wrist**		**0.285**	**0.445**	**0.296**	**0.310**
HildebrandLM^28^	Wrist	**60**	0.260	0.505	0.599	0.384
HildebrandNLM^29^	Wrist		0.263	0.493	0.632	0.395
Sasaki^25^	Hip		0.303	0.469	0.406	0.348
Freedson^17^	Hip		0.345	0.501	0.448	0.388
Kerr^7^	Hip		0.292	0.372	0.359	0.317
**Proposed**	**Wrist**		**0.268**	**0.307**	**0.310**	**0.281**

Significance bold: our proposed method.

**Table 5:** Repeated Measures ANOVA Results of EE estimation by related works.

Method	Window	F-statistic	Effect Size (d)	p-value
Crouter^32^	10	F(1,25) = 366.28***	-3.75	< 0.001
Ray^26^	12.8	F(1,25) = 4.66*	0.42	0.041
Staudenmayer^31^	15	F(1,25) = 59.60***	-1.51	< 0.001
Montoye^30^	30	F(3,75) = 56.93***	-2.12	< 0.001
Montoye^34^			-0.73	0.003
Lyden^33^			n.s.	> 0.05
HildebrandLM^28^	60	F(5,125) = 43.49***	-1.80	< 0.001
HildebrandNLM^29^			-2.33	< 0.001
Sasaki^25^			-1.38	< 0.001
Freedson^17^			-2.01	< 0.001
Kerr^7^			-0.85	0.001

Note: F-statistic represents the overall ANOVA test for each window size. Negative effect sizes indicate lower RMSE (better performance) for the proposed method compared to existing algorithms. *p < 0.05, ***p < 0.001, n.s. = not significant after Bonferroni correction.

To assess the potential impact of mirroring left-handed participants’ data, we conducted a sensitivity analysis comparing the results using the 60-second window size for left-handed-only (n=4), right-handed-only (n=23), and the combined sample. The overall RMSE values for the left-handed-only, right-handed-only, and combined samples were 0.306, 0.278, and 0.281, respectively, indicating no substantial differences in algorithm performance based on handedness.

**Table 6:** Summary of activities and behavioral features during free-living instances in which proposed method over and underestimated EE with respect to Kerr et al.’s algorithm^7^.

Activity State	No. of Instances	No. of Unique Participants	Behavioral Features
***Underestimation***			
Walking	87	13	Hands in pocket, gait, holding bags, holding phone steady
Walking, shopping	12	1	Pushing cart, holding bags
Sitting, public transit, phone	62	4	Holding phone steady
Sitting	33	4	Swiveling, adjusting posture
Sitting, fidgeting	29	5	Adjusting devices
Sitting, phone	16	9	Talking on phone, hand stationary near ear
Bathroom	2	1	Urinating (male), hands against wall
Talking	21	7	Slight body movements
Eating	17	4	Scrolling on phone
Camera Blocked	67	11	Unable to identify activities
***Overestimation***			
Sitting, phone	98	13	Scrolling, tapping on phone, gesticulating
Walking	23	6	Opening doors, arms dangling
Driving	10	3	Wheel turning, manipulation of dashboard
Walking, shopping	8	2	Reaching for items, adding items to cart/bag
Sitting, fidgeting	78	15	Dependent on type of activity being performed
Cleaning	13	4	Object manipulation
Phone, TV	34	4	Scrolling on phone, tapping on phone
Eating	9	3	Sitting, standing
Fidgeting	21	8	Smoking, drinking, device adjustment, sitting
Camera Blocked	20	6	Unable to identify activities

**Table 7:** Description of activities performed by participants in-lab.

Activity	Description	Intensity
Computer	Type on a computer while seated	Sedentary
Reading	Read a book or magazine while reclining	Sedentary
Lying down	Lying still, awake on the floor, doing nothing	Sedentary
Standing	Stand still on the ground while fidgeting	Light
Sweeping	Slow, light effort of sweeping the floor	Light
Walking slowly	Walk slow chosen pace on a treadmill	Light
Push-ups	Do push-ups against a door	Moderate
Walking normally	Walk average chosen pace on a treadmill	Moderate
Squats	With feet shoulder-width apart, flex knees 90 degrees and straighten legs again; repeat	Moderate
Walking fast	Walk fast chosen pace on a treadmill	Vigorous
General aerobics	Follow along with provided aerobics video	Vigorous
Step Test	Step up and down 0.25 m at a rate of 30 steps per minute	Vigorous

Each activity was performed for 5 minutes, followed by 5 minutes of rest.

When comparing the proposed method to each of the established methods, we ran the model using the window sizes proposed by each established method. Our method outperformed 6 of the 7 established methods at their respective window sizes. The method by Ray et al.^26^, which uses conditional random fields to estimate EE, slightly outperformed our method at their proposed window size of 12.8 seconds, yielding an RMSE of 0.301 compared with the RMSE of the proposed method (0.322). Nevertheless, the proposed method yielded low variability in RMSE (ranging from 0.281 to 0.333) compared with other established methods across a wide range of window sizes (Table 4). In summary, we achieved consistent results, with most of the difference between predicted METs and ground truth METs falling within 1.98 SDs from the mean when predicting light, sedentary, and moderate-to-vigorous activities (Fig. 2, 3, 4, 5, 6).

**Fig. 1:**
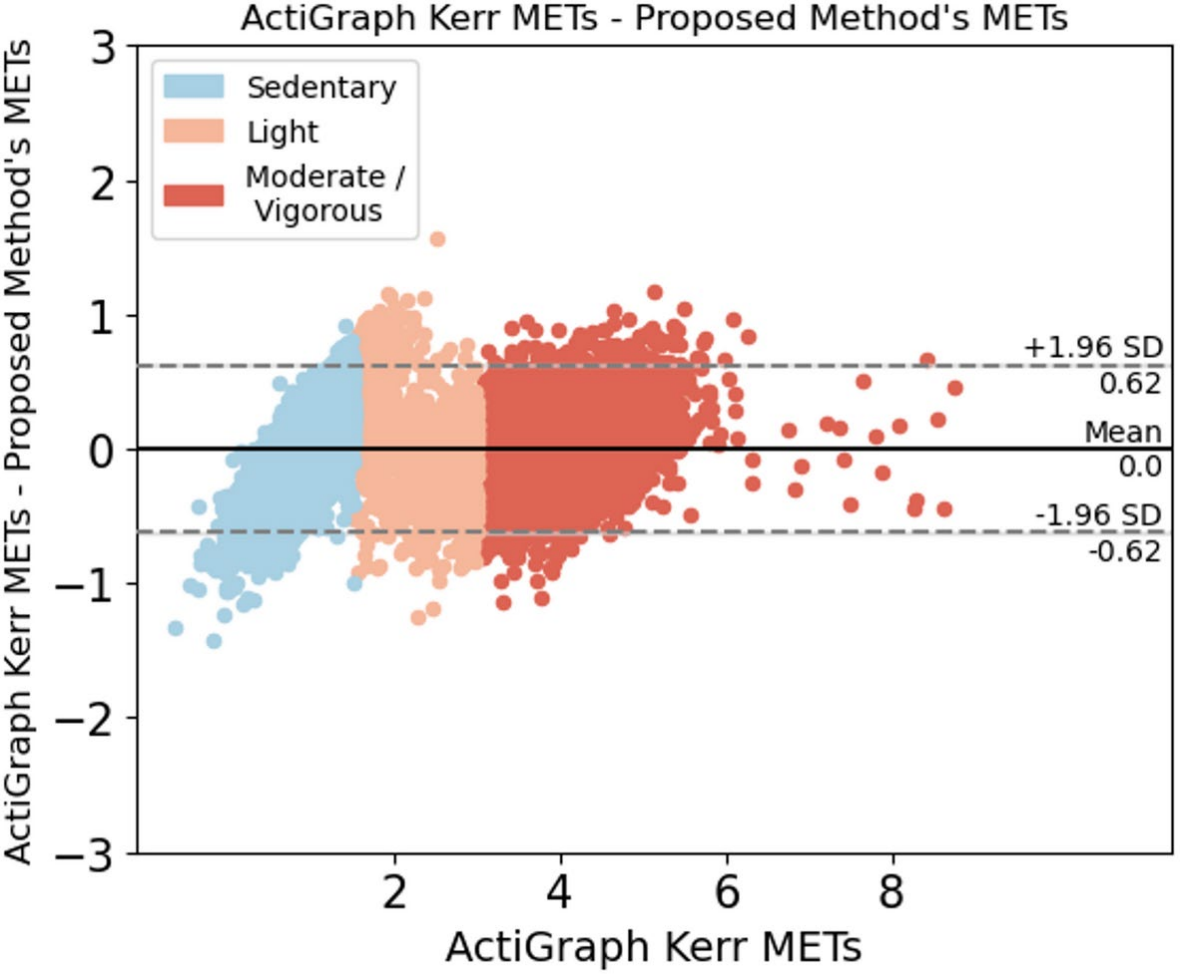
Bland-Altman plot for data collected in free-living conditions (n=14,045 minutes) showing the difference between the metabolic equivalent of task (MET) estimated by the Kerr et al.’s algorithm^7^ and the proposed method. Minutes are classified as sedentary, light, and moderate-to-vigorous physical activity based on Kerr et al.’s estimation^7^.

**Fig. 2:**
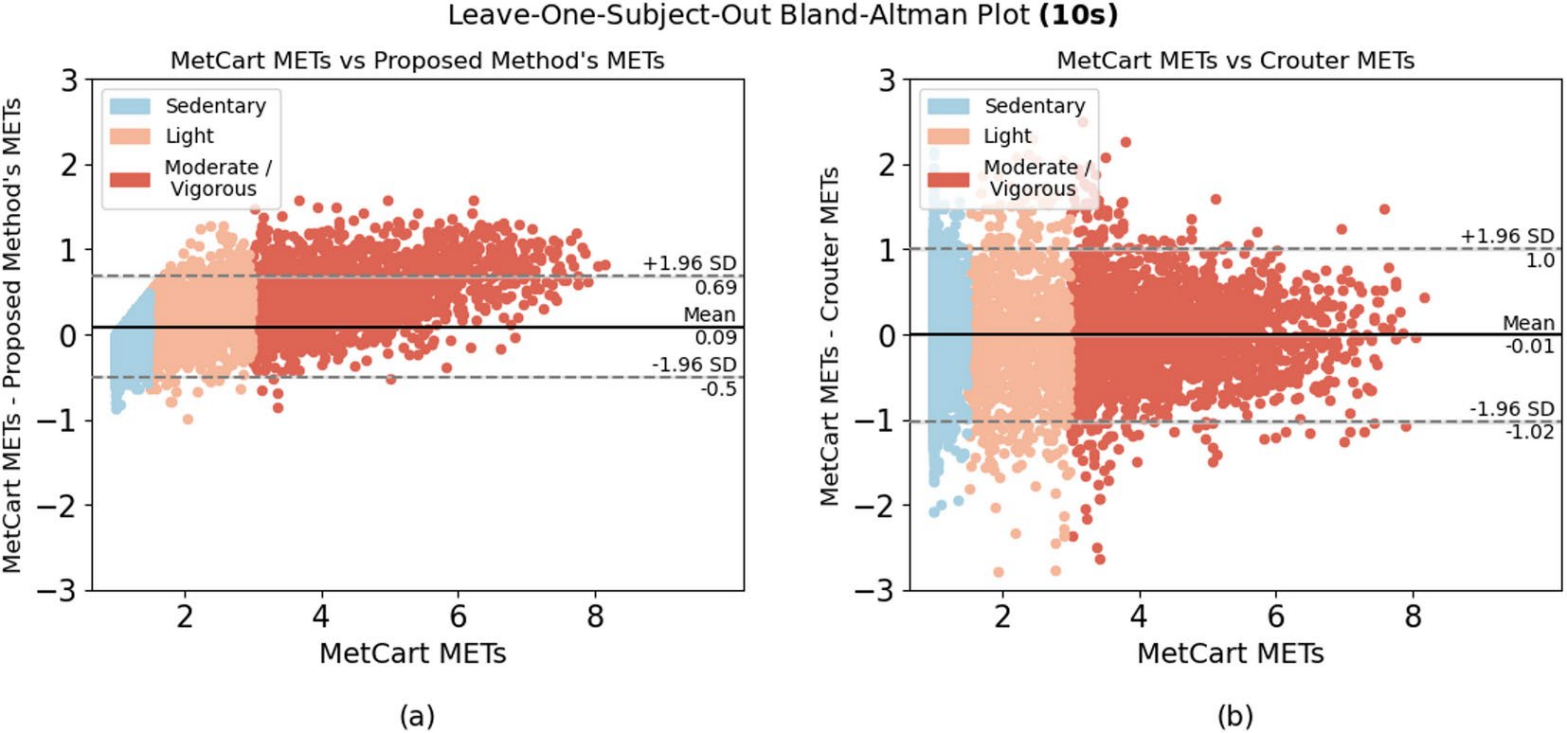
Bland-Altman plots for algorithms applied to activities performed in-lab using the 10-second window. Difference between (**a**) MetCart METs and METs estimated by our proposed method and (**b**) MetCart METs and Crouter et al. METs^32^. MET, metabolic equivalent of task.

**Fig. 3:**
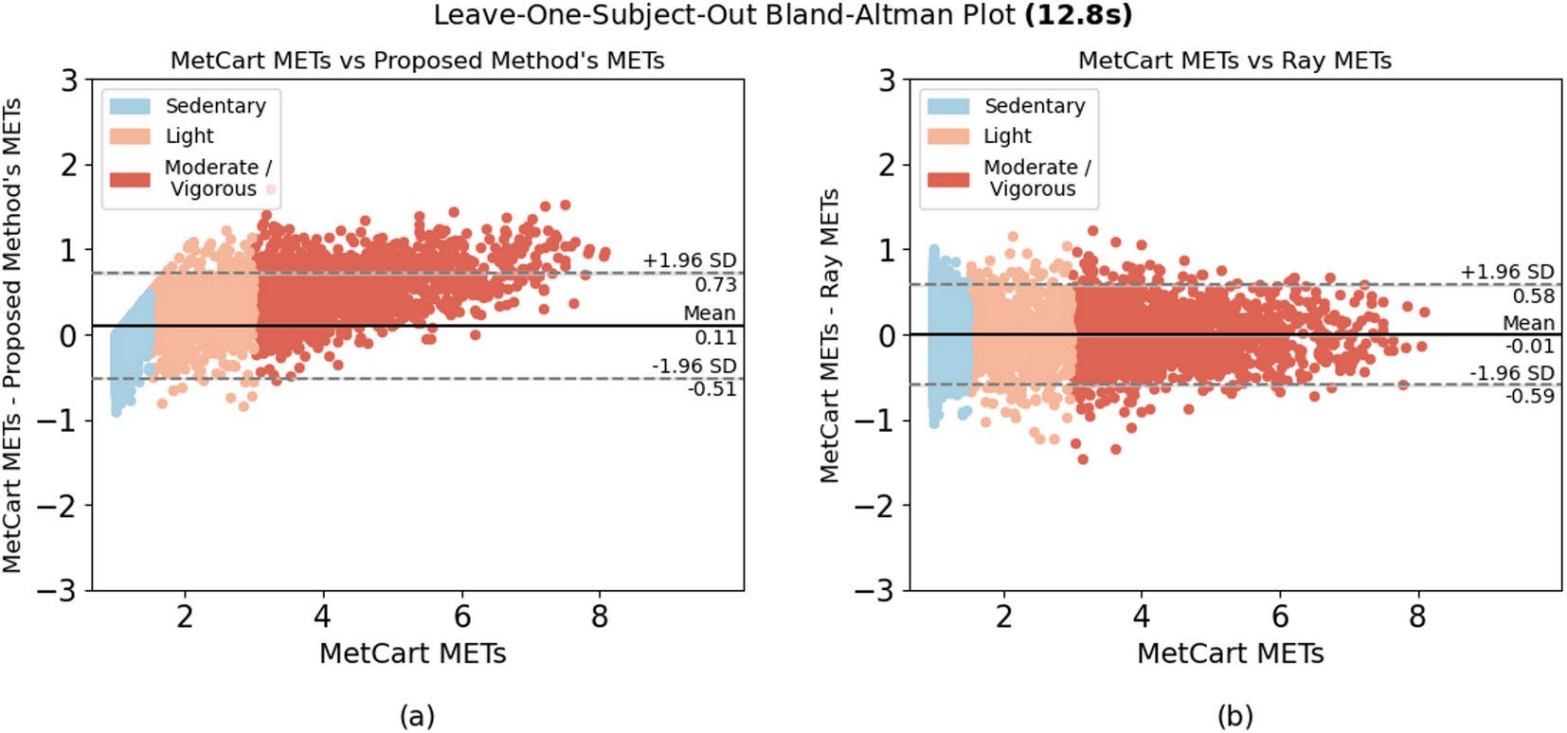
Bland-Altman plots for algorithms applied to activities performed in-lab by 12.8-second window. Difference between (**a**) MetCart METs and METs estimated by our proposed method and (**b**) MetCart METs and Ray et al. METs^26^. MET, metabolic equivalent of task.

### Statistical comparison of In-lab MET estimations

Within each window size, a repeated‑measures ANOVA comparing the algorithms revealed statistically significant differences at every window tested (Table 5). Effect sizes ranged from moderate to large (|d| = 0.42 to 3.75), with the strongest effects observed at the 10-second window compared to Crouter’s method (d = -3.75) and at the 60-second window compared to existing methods (|d| = 0.85 to 2.33). The only exception was at the 12.8-second window, where Staudenmayer’s method showed slightly better performance (d = 0.42). At the 30-second window, while differences with Montoye’s methods were significant, the comparison with Lyden’s method did not reach statistical significance after Bonferroni correction.

### Free-living MET evaluation

We enrolled 25 participants in the free-living study. To ensure high data quality, we established minimum data requirements: participants needed to wear devices for at least 80% of waking hours (approximately 12-14 hours per day) with complete, unobstructed data from the smartwatch, ActiGraph, and wearable camera. Six participants were excluded due to critical data collection issues: 1 participant was excluded due to complete data collection failure across all devices; 2 participants had critical data upload malfunctions resulting in deleted or missing wrist data; 2 participants collected insufficient wrist data (less than 30 minutes) due to data collection app crashes; 1 participant was excluded due to insufficient device connectivity and incomplete data. For the remaining 19 participants, we first segmented the camera footage into 60-second segments and ran the proposed MET estimation model. We then visually inspected the footage to identify activity types and behavioral features (Table 6). Out of 14,045 minutes of free-living data, we found 346 underestimated minutes and 314 overestimated minutes of METs prediction (660 minutes total; 4.7% of all minutes collected) when comparing our proposed model with Kerr et al.’s model^7^ (Fig. 1). Any minute for which the difference between the two algorithms exceeded ±1.96 SD from their mean difference was classified as either underestimated or overestimated. 95.03% of minutes fell within an acceptable estimation range. After removing minutes during which the wearable camera was obstructed, 573 minutes of over and underestimations remained, all of which were then subjected to visual inspection. After visual inspection of these minutes, we classified the observed activities into 9 categories, summarized the behavioral features of each activity type, and assigned compendium-based MET values to them as ground truth. Our proposed algorithm achieved an RMSE of 0.294, compared to 0.309 for Kerr et al.’s algorithm, further demonstrating the algorithm’s improved performance in estimating energy expenditure across real-world activities. We found walking and talking on the phone were among the most prevalent activities for which we underestimated METs, whereas scrolling/typing on the phone, and fidgeting were among the most prevalent activities for which we overestimated METs. Sitting activities demonstrated varied estimation errors depending on upper body movement and context. Sitting with minimal movement (e.g., watching TV) tended to be underestimated, while sitting with active upper body movements such as phone scrolling, gesturing, or device interaction led to overestimation of energy expenditure.

**Fig. 4:**
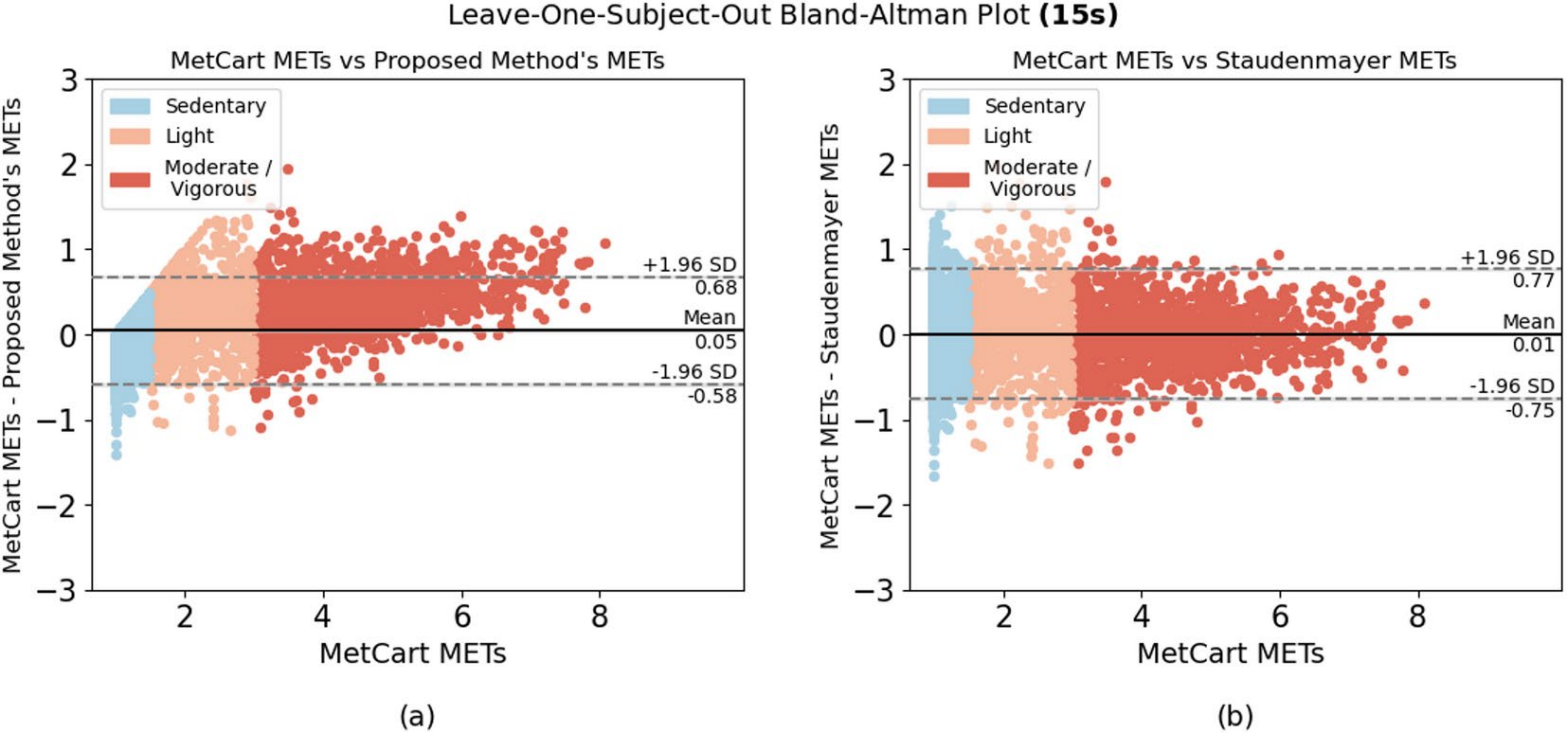
Bland-Altman plots for algorithms applied to activities performed in-lab using the 15-second window. Difference between (**a**) MetCart METs and METs estimated by our proposed method and (**b**) MetCart METs and Staudenmayer et al. METs^31^. MET, metabolic equivalent of task.

**Fig. 5:**
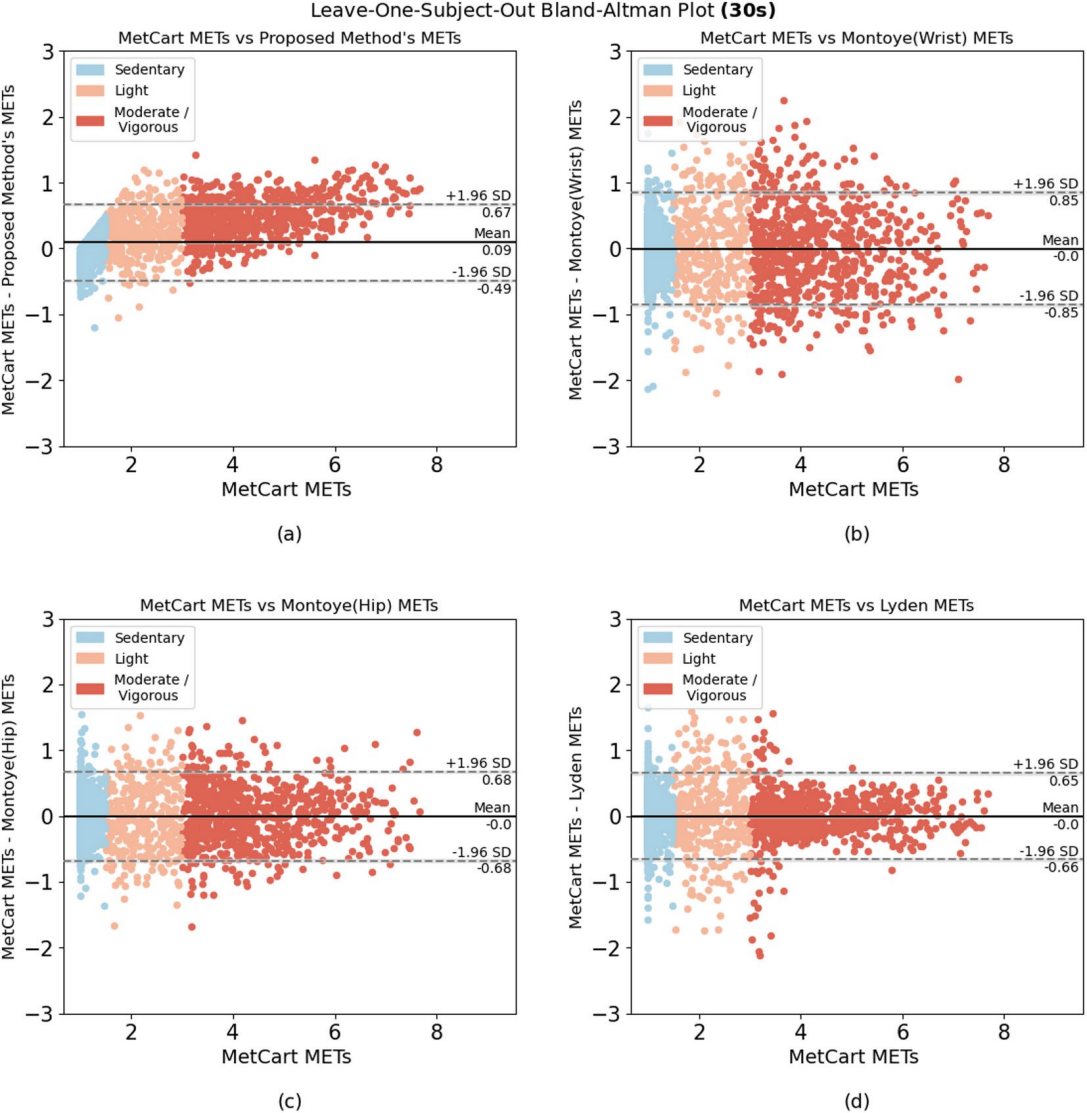
Bland-Altman plots for algorithms applied to activities performed in-lab using the 30-second window. Difference between (**a**) MetCart METs and METs estimated by our proposed method, (**b**) MetCart METs and Montoye et al. METs^34^, (**c**) MetCart METs and Montoye et al. METs^30^, and (**d**) MetCart METs and Lyden et al. METs^33^. MET, metabolic equivalent of task.

**Fig. 6:**
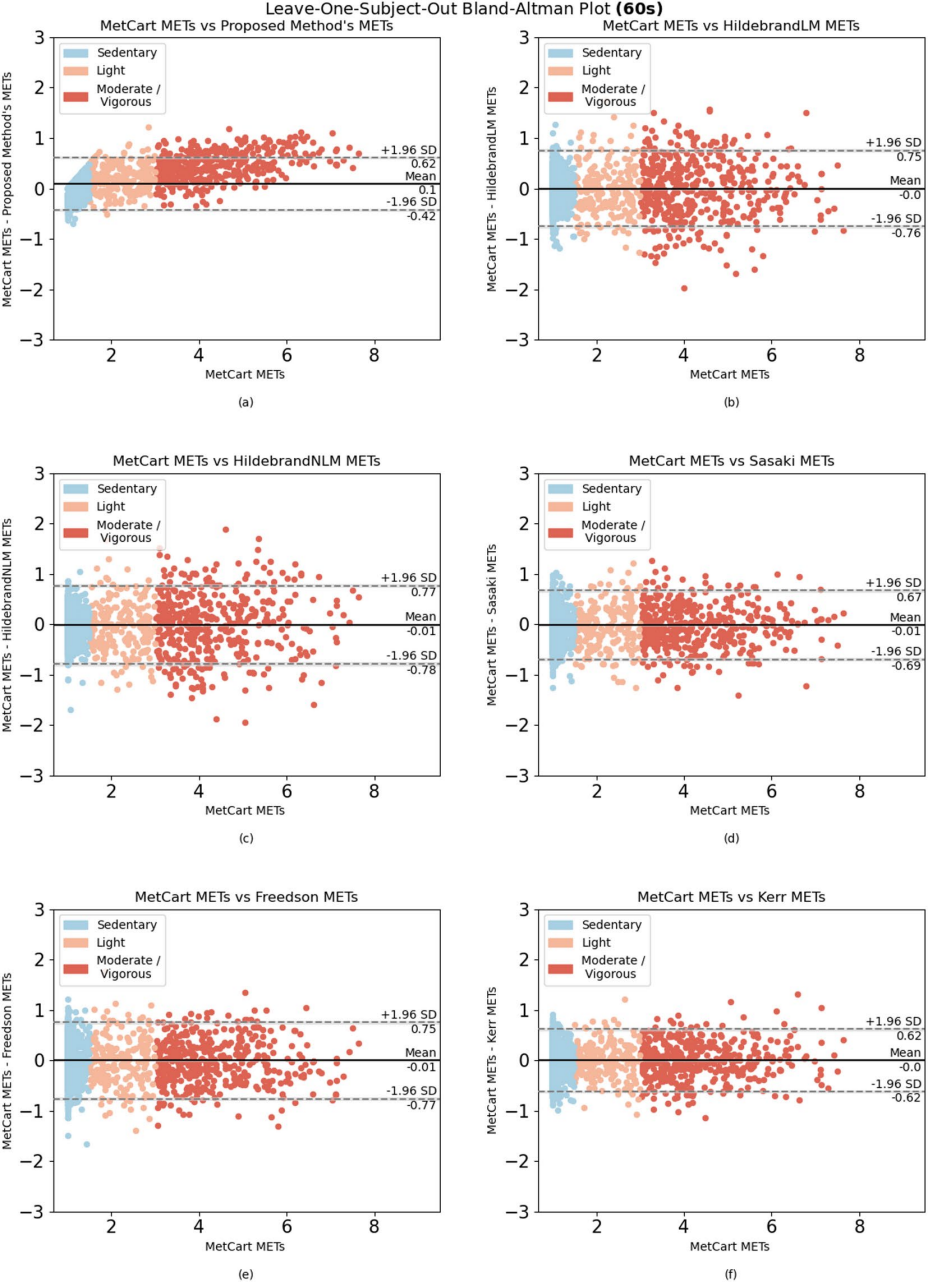
Bland-Altman plots for algorithms applied to activities performed in-lab using the 60-second window. Difference between (**a**) MetCart METs and METs estimated by our proposed method, (**b**) MetCart METs and Hildebrand linear model METs^27,28^, (**c**) MetCart METs and Hildebrand nonlinear model METs^29^, (**d**) MetCart METs and Sasaki et al. METs^25^, (**e**) MetCart METs and Freedson METs^17^, and (**f**) MetCart METs and Kerr et al. METs^7^. MET, metabolic equivalent of task.

**Fig. 7:**
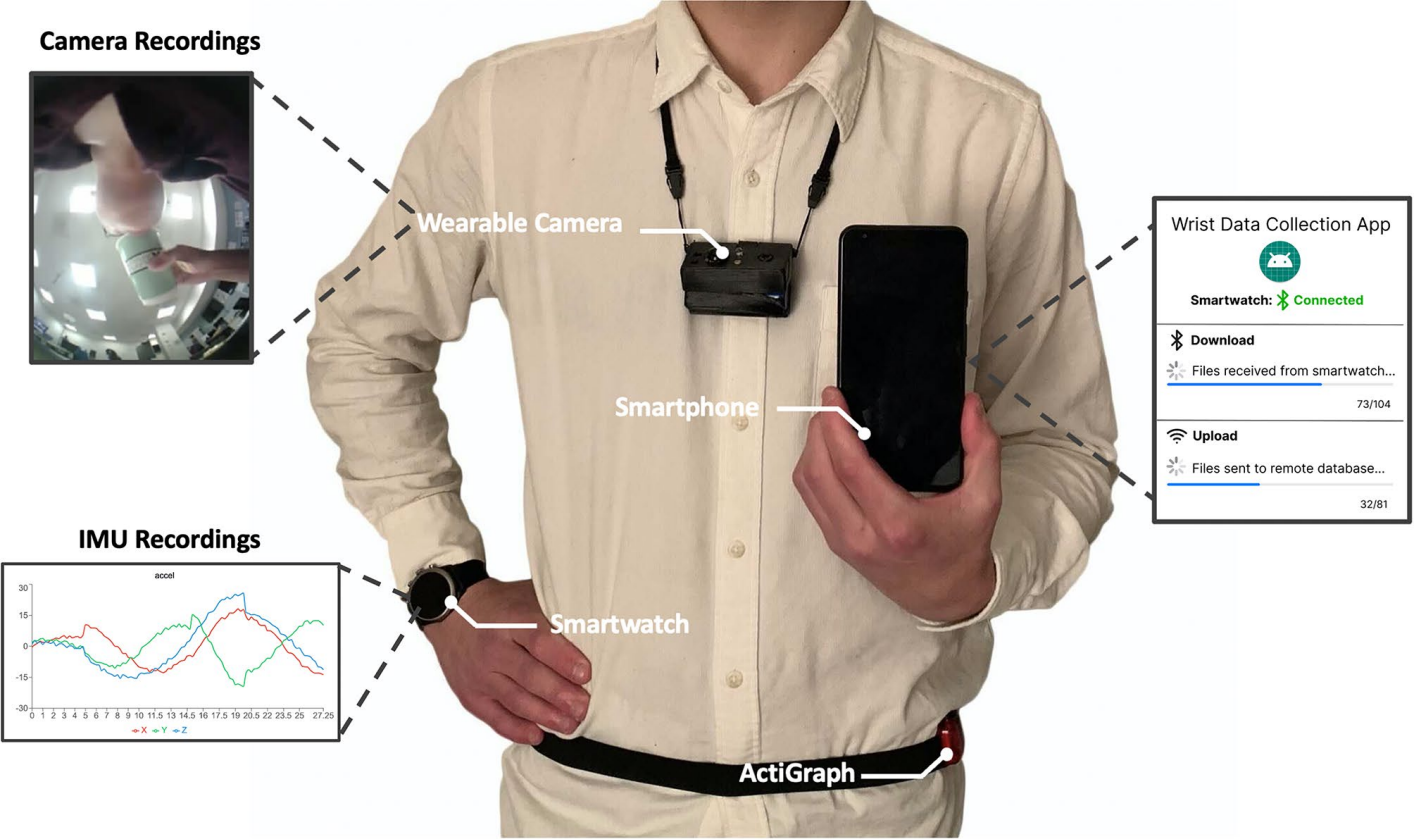
Devices used in the study: A wearable camera was used to provide visual confirmation of human behavior performed in the wild. We utilized the smartwatch tri-axial accelerometer and gyroscope to estimate energy expenditure using our proposed algorithm and compared it against multiple algorithms^7,17,25,26,32–34^ using the hip-worn ActiGraph.

## Discussion

To date, many works have examined the validity of proprietary algorithms from commercial activity monitors^7,17,25–34^. However, to the best of our knowledge, none of them have published an open-source algorithm that others can replicate using raw data obtained from commercially available wrist-worn sensing devices. Our proposed method is not only highly accessible, portable, and transparent, but it also addresses the crucial need for wrist‑mounted IMU algorithms to estimate energy expenditure—leveraging a placement that typically yields higher tracker adherence than hip‑mounted devices^13^. We offer open-source software enabling researchers to calculate METs from wrist‑mounted IMU data, and we also share our dataset so they can build and test new machine‑learning and regression models for estimating energy expenditure in people with obesity.

Our approach is similar to that of Crouter et al.’s design of a 2-regression equation^32^, which seeks to first classify whether the behavior is ambulatory or non-ambulatory and then applies the specified algorithm to estimate EE. Although the original 2-regression equation was introduced in 2006, recent comparisons with highly sophisticated algorithms show that the 2-regression method performs well in distinguishing between ambulatory and non-ambulatory states^35^. Our findings further support the approach of first classifying the activity type and then building a type-specific regression model to estimate METs. Statistical analysis confirmed that our proposed method demonstrated significantly lower root mean square error compared to most existing research-grade algorithms^34^, providing robust evidence of its improved performance in energy expenditure estimation. The improvement likely arises from the method’s larger sliding window, which captures longer portions of each activity and therefore characterizes and classifies them more effectively.

A common concern in EE estimation research is the comparability and usability of accelerometry signals recorded from different body locations^13^. While uniaxial wrist accelerometry has shown poor performance in EE estimation^36^, triaxial wrist accelerometry has performed well enough to suggest its viability as a reliable method of EE estimation. In this work, we retrained and validated the algorithms from related works using the same sliding window, data processing steps, and cross-validation as our proposed method. Although our method did not exceed the performance of Ray et al.’s hip-worn triaxial-accelerometer approach^26^, it achieved comparably low RMSEs for sedentary, light, and moderate-to-vigorous activities while using the more widely adopted wrist-mounted sensor form factor.

Wrist IMU data collection protocols typically require the sensing device to be worn on the non-dominant hand on the assumption that the non-dominant hand–which is generally not used in wrist-intensive fine motor activities (e.g., writing, operating machines, gesturing)–is the hand most representative of gross body movement. However, recent literature has shown that the choice of wrist (dominant vs non-dominant) does not affect the accuracy of EE estimates^2,23^. In addition to providing data useful to EE estimation, mounting an IMU device on the dominant hand enables EE estimation to occur alongside other important activity recognition tasks that assess behavioral features beyond the types and intensities of physical activities, such as the occurrence of eating, drinking, and smoking; all of which relate to caloric intake. Thus, dominant-hand wrist IMU could conceivably underpin a monitoring system capable of estimating both the intake and expenditure of caloric energy, therefore enabling a composite estimation of overall energy balance.

In our in‑lab study of 27 participants with obesity—each wearing wrist‑ and hip‑mounted sensors alongside a metabolic cart—our algorithm achieved the smallest error in energy‑expenditure estimates compared against the metabolic cart, outperforming all hip‑worn ActiGraph‑based algorithms at the 60‑second window size. Since ground truth EE varies when employing different window sizes, we test 9 window sizes ranging from 5 to 90 seconds. Of the window sizes tested, our proposed method outperforms all other algorithms under the same settings except for the 12.8-second window size used by Ray et al.^26^. However, the best performance of the proposed method is achieved at the 60-second window size, which outperformed all other methods, including the model by Ray et al.^26^ at 12.8 seconds. The proposed method yields low RMSE across all window sizes. Given the other methods were optimized for a specific window size, we are unable to confirm their performance at alternative window sizes. We posit the reason being that our method, which consists of a two-step activity classification (sedentary vs. non-sedentary) and subsequent application of a regression model on non-sedentary activity segments, relies on features from relatively long periods of the window to achieve greater precision in estimating METs. The statistical features of accelerometer and gyroscope signals are more prone to error when analyses are constrained to shorter window sizes (such as 12.8 seconds), and the RMSSD and dominant frequency features of the accelerometer signal are generally more stable as reflections of physical activity intensity over extended periods. We suspect our proposed method achieved its best performance at 60 rather than 90 seconds because activity pace (i.e., intensity) often shifts considerably over 90 second intervals, in contrast with activity state, which frequently persists beyond intra-activity intervals of consistent activity pace. Our visual inspection of instances in which the model over and underestimated METs suggests that, as a rule of thumb, the proposed method underestimates EE when the dominant hand is underactive relative to the rest of the body (e.g., holding a phone static against one’s ear while walking) and, by the same token, overestimates EE when the dominant hand is overactive relative to the rest of the body (e.g., playing a game on a phone while sitting still).

In our free-living study, a greater number of walking minutes are exhibited in underestimation (99 instances) compared with overestimation of EE (31 instances). We also find that when sitting, estimation error depends heavily on the type of activity being performed. Sitting on public transportation, which is often concurrent with cell phone use, results in underestimation of the proposed algorithm. We suspect that participants “stabilize” their dominant hands (in which their phones are held) in order to read the screen, reducing the influence of a train or bus movement that is fully present in the hips. Wrist stabilization also appears in other activities, such as co-occurrences of walking and phone use, in which the wrist is held stable to the ear while the hips move freely, leading to underestimation of EE from wrist-worn sensors. Shopping is another activity in which estimation error depends on whether individuals are actively selecting and examining objects, have their hands stationary on the shopping cart, or are holding a weighted bag. Specific activities have more readily apparent causes of estimation error, such as phone use while sitting (overestimation), cleaning (overestimation), and swiveling in a chair while keeping hands on a keyboard or document (underestimation). Further research is needed to investigate how to use context obtained from other sensors to further optimize the determination of EE using the wrist-worn device. One insight is to use an integrated camera to detect activity context and/or environmental influences. Lanyard-worn wearable cameras^37^ help to explain the context of activities that an inertial-based sensor would struggle to quantify. In a similar vein, wrist-mounted cameras^38^ have shown promising results for inferring daily activities from detected objects-in-hand. We aim to further explore the utility of additional concurrent sensing modalities to refine MET estimations in light of their associated behavioral contexts.

## Conclusion and future work

In this study, we developed and validated a machine learning algorithm to estimate energy expenditure from commercial smartwatch data in people with obesity. In our in-lab protocol, which included 2,189 minutes of data from 27 participants, our proposed method achieved a root mean square error (RMSE) of 0.289 METs at the 60-second window size, outperforming most existing algorithms. Statistical analysis provided robust validation of our algorithm’s performance. The repeated measures ANOVA revealed statistically significant differences between our proposed method and established algorithms. Particularly notable were significant differences at the 10-second window compared to Crouter’s method (effect size $d = -3.75$) and at the 60-second window across various existing methods (effect sizes $|d| = 0.85$ to 2.33).

In the free-living study, involving 14,045 minutes of data from 25 participants, our proposed method estimates were within $\pm 1.96$ SD of the best actigraphy-based estimates for 95.03% of minutes. These results demonstrate the potential for commercial wrist-worn devices to reliably measure energy expenditure in people with obesity when combined with population-specific models and contextual information from wearable cameras. However, it is important to acknowledge the limitations of our validation approach, which may have favored our algorithm due to the specific population and consistent study setting. While our findings highlight the benefits of developing models tailored to specific populations, they should not be interpreted as evidence of absolute superiority over existing algorithms.

Future work should focus on further refining the proposed algorithm, incorporating additional sensing modalities, and validating it in larger, more diverse samples across different settings and populations. Moreover, incorporating compendium estimates with our camera ground truth could enable additional free-living validation, which we will consider for future studies. We also plan to investigate how activity variability, beyond binary classification, might improve energy expenditure estimation accuracy. These further investigations will help establish the generalizability and robustness of the proposed method and provide a more comprehensive understanding of its performance. Despite these limitations, our study underscores the promising role of commercial wrist-worn devices in advancing the accurate and ubiquitous monitoring of energy expenditure in people with obesity.

## Method

### Participants

Participants were recruited in two phases for the in-lab and free-living studies via flyers, Craigslist, and ResearchMatch services. A separate cohort of participants was recruited for each study. Potential participants were screened against our inclusion criteria ($\ge$18 years of age, body mass index [BMI] $\ge$30 $kg/m^2$), informed of study procedures, and scheduled to visit the laboratory. Study procedures were approved by the Institutional Review Board at Northwestern University (STU00208545) and performed in accordance with the Declaration of Helsinki. In addition, all participants provided written informed consent before participation at the start of the first visit. Following consent, research staff measured participants’ height and weight using a standard analog scale (Detecto mechanical eye-level physician scale with height bar) to confirm BMI $\ge$30.

### Study devices

#### Smartphone, smartwatch, and data gathering app

Participants donned an LG Nexus 5 smartphone (LG Electronics, Seoul, South Korea) and a paired Fossil Sport 43-mm smartwatch Gen 4, model number: FTW4019 (Fossil Group Inc., Richardson, TX) to capture wrist activity (Fig.7). We designed a data-gathering application to run on the smartwatch and collected raw accelerometer and gyroscope data from the watch’s inertial measurement unit (IMU) at a sampling rate of 20 Hz. The smartwatch was worn on the participant’s dominant wrist during waking hours to ensure contact with the skin, and to better capture energy expenditure during daily activities, even when they involve fine motor control and object manipulation, which are predominantly performed with the dominant hand. Wearing two wrist-worn devices is often infeasible in longitudinal studies, and many studies recommend placing the device on the dominant-hand to capture important daily activities related to eating, writing, or using common household objects. As a result we intentionally wanted to assess how well we can capture the energy expenditure of daily activities from the dominant hand. If they were left-handed, the IMU data collected was adjusted to mirror that of the right hand, following the approach by Kyritsis et al.^39^ The raw sensor data were stored on the watch’s internal memory. Once the phone and watch were connected to their respective chargers, the smartwatch application transferred the raw data from the watch to the phone’s internal storage via Bluetooth. The phone then transferred data over Wi-Fi to a secure server from which the study team could retrieve it. The code for this application is maintained in a Git repository by the study team and is available to the research community upon request.

#### ActiGraph accelerometer

Participants wore an ActiGraph GT3X+ (ActiGraph Corp., Pensacola, FL) device on the hip contralateral to their dominant hand (Fig.7). The ActiGraph was set to record data at 60 Hz with idle sleep mode disabled. ActiGraph data was downloaded and aggregated into specified epochs to compare with the related works using ActiGraph’s proprietary ActiLife software.

#### Wearable camera

A chest-worn wearable camera was used in the free-living portion of this study to document activities performed (Fig.7). The camera was built around an ARM-Cortex M4 microcontroller and comprised a dual-stream red/green/blue (RGB) camera (OmniVision OV2640) and a low-cost, low-power 8x8 infrared (IR) thermal camera (Panasonic GridEye 8x8). The device also contains a charging and battery management subsystem and an onboard microSD card to store the dual-stream RGB and IR sensor data. The wearable camera is affixed to the body using 2 methods: 1) a lanyard is worn around the neck to fix the vertical position of the device, and 2) a detachable magnetic backplate is placed behind the user’s shirt to anchor the device to the chest, preventing it from swinging as the wearer moves. The device is equipped with an IR sensor and an RGB camera with a $180^{\circ }$ fisheye lens, enabling capture of both the wearer’s activities and contextual features of their environment. The sensor and lens point upward from their position on the chest, providing a view of the face and upper torso, as well as a limited view of the surrounding area. This view allows for positive identification and validation of free-living behaviors^40^. The camera collects raw RGB video and IR data streams at a sampling rate of 5Hz, encrypting them in real time using a stream cipher (salsa20), and stores the encrypted images onto the onboard microSD card. Data are later downloaded and decrypted by the study team for use in activity classification and validation.

### Data collection procedures

#### In-lab study

After equipping the wearable devices, participants were asked to perform a series of 12 activities, each 5 minutes long, with 5-minute rest periods between each activity. Activities were carefully selected to provide a range of both physical intensities and activity domains (including sedentary, light, moderate, and vigorous; leisure, transportation, household, occupational; Table 7)^41^). If participants were unable to perform an activity for 5 minutes, they could opt out of performing said activity or simply stop anytime during the 5-minute duration. While completing the 12 activities, participants were fitted with a Hans Rudolph 7450 Series V2 oronasal mask connected to a Vmax 29n Encore metabolic cart (Vyaire Medical) to record breath-by-breath pulmonary gas exchange. We chose the metabolic analyzer as our criterion because it directly measures energy expenditure, capturing individual variations that standardized compendium values may not reflect in people with obesity. Following standard gas and flow calibration of the machine, participants completed all activities within the physical space allowed by the stationary machine. Collected values of oxygen consumption (VO2) and carbon dioxide production (VCO2) were then used to calculate energy expenditure using the Weir equation^42^:

$$EE (kcal/min) = 3.941 \cdot VO2 (L/min) + 1.106 \cdot VCO2 (L/min)$$

METs were subsequently calculated by dividing the energy expenditure by 3.5 $ml \cdot kg^{-1} \cdot min^{-1}$ for each participant’s activities.

To avoid temporal concentration of any one intensity level, the activities are ordered as follows to disperse exertion evenly across the study duration: computer, walking slowly, walking normally, standing, squats, reading, general aerobics, sweeping, push-ups, walking fast, lying down, and step test (Table 7 provides descriptions of the activities^41^).

#### Free-living study

We recruited a different set of participants for the 2-day free-living study. These participants were trained to use the devices and then asked to wear the smartwatch, ActiGraph, and wearable camera while performing their typical day-to-day activities within their homes or other usual environments. Participants were instructed to take off and charge the devices when they go to bed and to put the devices back on when they wake up the next morning. After 2 days, participants returned the devices to study staff.

#### Visual confirmation of free-living activity

To classify activities performed in the free-living condition, we established ground truth (i.e., incontrovertible evidence of the presence of a given behavior) with footage from the wearable camera. This allowed us to visually confirm participants’ activities at a given moment and to compare wrist and ActiGraph data streams to the activity (or inactivity) that produced them. We extracted and processed the data streams when participants returned the devices. Specifically, we segmented the videos by 60-second windows with 50% overlap and imported them into ELAN, an annotation tool for video recordings^43^. An annotator then provided labels to summarize activity types and behavioral features seen in the video. Two annotators then identified similarities across labels and reached a consensus on a final set of labels through discussion with the authors. If there is a discrepancy between annotators, the label is resolved by presenting the footage to the team for a majority vote.

### Data analysis: proposed algorithm

In order to develop an EE algorithm, we designed a pipeline that predicts METs for various time durations (i.e., overlapping windows with 50% overlap) ranging from 5 to 90 seconds (ranges based on prior literature^7,17,25,26,32–34^) using accelerometer and gyroscope data from the commercial smartwatch. Accelerometer and gyroscope data were first synchronized with the METs values for each activity, and we removed the first 2 minutes of data at the beginning of each activity to ensure the stability of VO2 measurement. The ground truth MET value, sampled initially at 20 Hz, was then aggregated accordingly to match the selected window size. Generating the proposed method’s MET predictions involved 2 main steps: 1) classification of sedentary and non-sedentary activities and 2) application of a regression model to the non-sedentary activities to estimate METs. Activities classified as sedentary were assigned a MET value of 1.0 based on the distribution of EE across all sedentary activities, and a regression model was used to predict MET values for non-sedentary activities. Regression-derived MET estimates for non-sedentary activities that fell below 1.0 were reset to 1.0, the value corresponding to the resting metabolic rate during quiet sitting.

#### Classification model

The first stage of the pipeline consists of a binary classification of each window into sedentary or non-sedentary activity classes. In our experiment, sedentary activities consisted of resting, typing on a computer, reading, and lying down (Table 7). For each of the 6 axes, we incorporated 42 descriptive statistical features that are widely used^44^ for activity recognition, including median, mean, maximum, minimum, range, standard deviation, and root mean square.

We tested multiple machine learning models, including Random Forest, Support Vector Machines (SVM), eXtreme Gradient Boosting (XGBoost), and K Nearest Neighbor. The XGBoost classifier outperformed all classifiers in classifying activities into sedentary/non-sedentary activities. Gradient boosting has shown success in a wide range of machine learning applications^45,46^. Gradient boosting machines produce competitive, highly robust, and interpretable classifications^47^. The XGBoost algorithm is sufficiently robust to support fine-tuning, and its regularized model formalization offers reasonable control against overfitting^48^. Recent studies also have demonstrated XGBoost’s effectiveness in activity recognition, such as detecting physical activity types^49^ and classifying daily physical behaviors^50^.

#### Regression model

After initial binary classification, we extracted intensity-based features from windows classified as non-sedentary and incorporated them into the regression along with demographic information. We then applied a wrapper-based feature selection algorithm^51^ to select the optimal subset of features. Before feature extraction, we applied L2 normalization to smooth the signals within each window. Demographic features included in the analysis are participant age, sex, weight, height, and BMI, as well as the interactions between these features. Intensity-based features used in prior activity recognition literature were extracted from the accelerometer signal and comprised $K_m$ motion estimates^52^, root mean square of successive differences (RMSSD)^53^, and dominant frequency^54^. We found these types of activity intensity-based features important to our estimations because accelerometry signals of different activity types display substantial variation in their successive difference patterns. For instance, accelerometer data shows more frequent changes during high-intensity activities than low-intensity activities, which is why these features were selected.

$K_m$ motion estimates of accelerometer data segments are calculated by the Panasonic equation^52^ and aggregated based on the selected window size (Eq.1). RMSSD is calculated with the equation shown in Equation 2, in which successive differences between adjacent data points were calculated, squared, and averaged. To estimate the dominant frequency, a Butterworth bandpass filter with a passband between 0.3-8 Hz was applied, consistent with the Nyquist theorem and the typical frequency range of human movement (0.3-3.5 Hz). This filtering approach ensures accurate extraction of dominant frequency features from the accelerometer signal, followed by the Fast Fourier Transform (Eq. 3). $x_i$, $y_i$, and $z_i$ represent the values of the three axes of accelerometer data at time point *i*, and *n* is the number of data points.

1 $$\begin{aligned} K_m= & \sqrt{\frac{1}{n-1}[(\sum _{i=0}^{n} x_i^{2} + \sum _{i=0}^{n} y_i^{2} + \sum _{i=0}^{n} z_i^{2} ) - \frac{1}{n} ((\sum _{i=0}^{n} x_i)^{2} + (\sum _{i=0}^{n} y_i)^{2} + (\sum _{i=0}^{n} z_i)^{2})] } \end{aligned}$$

2 $$\begin{aligned} RMSSD= & \sqrt{\frac{1}{n}\sum _{i=0}^{n} {({x_{i} - x_{i+1}})}^2 + {({y_{i} - y_{i+1}})}^2 + {({z_{i} - z_{i+1}})}^2 } \end{aligned}$$

3 $$\begin{aligned} Freq(dominant)= & \underset{x}{\textrm{argmax}}|\sum _{i=0}^{n-1} {e^{-2 \pi j \frac{k i}{n}}({x[i]})}| \end{aligned}$$

### Evaluation

#### In-lab setting

For the in-lab experiment, we split the training and testing data by leave-one-participant-out cross-validation (LOPO-CV) and trained a random forest regressor to estimate METs. The model was evaluated in two parts: classification metrics including precision, recall, and F1 score for the binary classifier in distinguishing between sedentary and non-sedentary activities; and root mean square error (RMSE) for the regression model in predicting METs, compared against METs generated from the metabolic cart under LOPO-CV for each selected window size. We evaluated our proposed method using window sizes ranging from 5 to 90 seconds and compared the errors of our algorithm against 11 other established methods in the literature, for which code was either publicly available or the methods were sufficient enough for reproduction^7,17,25–34^. For methods that do not directly estimate the METs, we modified the models’ output to estimate METs directly. For instance, we adapted the two-level behavior classification (TLBC) algorithm originally proposed by Kerr et al.^7^ to estimate METs directly. The original algorithm used a combination of a random forest classifier and a hidden Markov model to predict four behaviors. For each of the established methods, we implemented and trained our model using the window size from the original work and generated Bland-Altman plots for both the established method and our proposed method against METs generated from the metabolic cart.

#### Statistical analysis of in-lab estimations

To rigorously assess whether the observed differences in RMSE between our proposed algorithms and related works were statistically significant, we conducted Repeated Measures ANOVA separately for each window size, as each window size involved different sets of comparison algorithms. Within each window size for each participant, we calculated the RMSE across all activities for each algorithm. This approach treats each algorithm's prediction as a within-subjects factor, with participants as the repeated measure. When ANOVA results suggest significant differences $(p < 0.05)$, we performed post-hoc pairwise comparisons using paired t-tests with Bonferroni correction for multiple comparisons. Effect sizes were calculated using Cohen’s d to quantify the magnitude of differences between algorithms.

#### Free-living setting

To evaluate the proposed method’s congruent validity on METs estimation in a free-living environment, the proposed model was trained using the entire in-lab data set, with the optimal window size and the optimal hyperparameter settings learned from the LOPO-CV. Given that we were not able to establish a MET ground truth in the free-living setting, and our proposed method performed optimally at a window size of 60 seconds, for a suitable and fair comparison, we compared the output of our model to that of the best established models that performed best at the same window size. We classify minutes as underestimated or overestimated using a statistical threshold of ±1.96 standard deviations (SD) from the mean difference between our proposed method and the best established model. We then inspected the minutes that were under and overestimated, and assigned compendium-based MET values^41^ to them as ground truth. Subsequently, we report the RMSE of our and Kerr’s estimation based on the compendium-based MET values.

## Data Availability

The complete datasets, algorithm implementations, and data processing scripts generated during this study are publicly available. The anonymized dataset is archived on Zenodo: https://zenodo.org/records/14858226; all code and documentation can be accessed in our GitHub repository: https://github.com/HAbitsLab/WristBased-EE-Estimation.
